# Mediation Analysis to Untangle Opposing Associations of High-Dose Docosahexaenoic Acid With IQ and Bronchopulmonary Dysplasia in Children Born Preterm

**DOI:** 10.1001/jamanetworkopen.2023.17870

**Published:** 2023-06-09

**Authors:** Thomas R. Sullivan, Jacqueline F. Gould, Jana M. Bednarz, Andrew J. McPhee, Robert Gibson, Peter J. Anderson, Karen P. Best, Mary Sharp, Jeanie L.Y. Cheong, Gillian F. Opie, Javeed Travadi, Peter G. Davis, Karen Simmer, Carmel T. Collins, Lex W. Doyle, Maria Makrides

**Affiliations:** 1 SAHMRI Women and Kids, South Australian Health and Medical Research Institute, Adelaide, South Australia, Australia; 2 School of Public Health, Faculty of Health and Medical Sciences, University of Adelaide, Adelaide, South Australia, Australia; 3 School of Psychology, Faculty of Health and Medical Sciences, University of Adelaide, Adelaide, South Australia, Australia; 4 Discipline of Paediatrics, Faculty of Health and Medical Sciences, University of Adelaide, Adelaide, South Australia, Australia; 5 Neonatal Services, Women’s and Children’s Hospital, North Adelaide, South Australia, Australia; 6 School of Agriculture, Food and Wine, Waite Campus, University of Adelaide, Adelaide, South Australia, Australia; 7 School of Psychological Sciences, Turner Institute for Brain and Mental Health, Monash University, Melbourne, Victoria, Australia; 8 Clinical Sciences, Murdoch Children's Research Institute, Melbourne, Victoria, Australia; 9 King Edward Memorial Hospital, Subiaco, Western Australia, Australia; 10 Newborn Medicine, Centre for Neonatal Research and Education, University of Western Australia, Perth, Western Australia, Australia; 11 Newborn Research, Royal Women's Hospital, Parkville, Victoria, Australia; 12 Obstetrics and Gynaecology, University of Melbourne, Parkville, Victoria, Australia; 13 Neonatal Services, Mercy Hospital for Women, Melbourne, Victoria, Australia; 14 Department of Child Health, Neonatal Intensive Care Unit, Waikato Hospital, Waikato, Hamilton, New Zealand; 15 Newborn Services, John Hunter Children’s Hospital, Newcastle, New South Wales, Australia; 16 School of Medicine and Public Health, University of Newcastle, Newcastle, New South Wales, Australia

## Abstract

**Importance:**

High-dose omega-3 docosahexaenoic acid (DHA) supplementation of children born at less than 29 weeks’ gestation has been shown to improve IQ despite increasing the risk of bronchopulmonary dysplasia (BPD). Given that BPD is associated with poorer cognitive outcomes, it is unclear whether the increased risk of BPD with DHA supplementation is associated with decreased benefit to IQ.

**Objective:**

To investigate whether the increased risk of BPD with DHA supplementation was associated with diminished IQ benefit.

**Design, Setting, and Participants:**

This cohort study used data collected from a multicenter, blinded, randomized controlled trial of DHA supplementation in children born at less than 29 weeks’ gestation. Participants were recruited from 2012 to 2015 and followed up until 5 years’ corrected age. Data were analyzed from November 2022 to February 2023.

**Interventions:**

Enteral DHA emulsion (60 mg/kg/d, to match the estimated in-utero requirement) or a control emulsion from the first 3 days of enteral feeds until 36 weeks’ postmenstrual age or discharge home.

**Main Outcomes and Measures:**

Physiological BPD was assessed at 36 weeks’ postmenstrual age. IQ was assessed at 5 years’ corrected age using the *Wechsler Preschool and Primary Scale of Intelligence, 4th Edition*; children from the 5 highest-recruiting Australian hospitals were assessed. The total effect of DHA supplementation on IQ was divided into direct and indirect effects using mediation analysis, with BPD as the presumed mediating variable.

**Results:**

Among 656 surviving children from hospitals involved in IQ follow-up (mean [SD] gestational age at birth, 26.8 [1.4] weeks; 346 males [52.7%]), there were 323 children with DHA supplementation and 333 children in the control group. Mean IQ was 3.45 points (95% CI, 0.38 to 6.53 points) higher in the DHA group than the control group, despite an increase in the risk of BPD (160 children [49.7%] vs 143 children [42.8%] with BPD). The indirect effect of DHA on IQ via BPD was not statistically significant (−0.17 points; 95% CI, −0.62 to 0.13 points), with most of the effect of DHA on IQ occurring independently of BPD (direct effect = 3.62 points; 95% CI, 0.55 to 6.81 points).

**Conclusions and Relevance:**

This study found that associations of DHA with BPD and IQ were largely independent. This finding suggests that if clinicians supplement children born preterm with high-dose DHA, any resulting increase in BPD risk would not be associated with meaningful reductions in the IQ benefit.

## Key Points

**Question:**

Is the increased risk of bronchopulmonary dysplasia (BPD) from high-dose docosahexaenoic acid (DHA) supplementation in the neonatal period associated with changes in benefits to IQ?

**Findings:**

In this cohort study involving 656 children born at less than 29 weeks’ gestation, the increased risk of BPD from neonatal DHA supplementation was not associated with a change in the 3.45-point benefit in IQ at 5 years’ corrected age.

**Meaning:**

This finding suggests that if clinicians supplement children born preterm with high-dose DHA, resulting increases in BPD risk would not be associated with meaningful reductions in the benefit to IQ.

## Teaser

This cohort study investigates the association of increased bronchopulmonary dysplasia risk from high-dose docosahexaenoic acid with decreased benefits to IQ among children born preterm.

## Introduction

Children born at less than 29 weeks’ gestation are at high risk of bronchopulmonary dysplasia (BPD) and have an IQ a mean 12 points lower than children born at term.^1,2,3^ The omega-3 (or n-3) polyunsaturated fatty acid docosahexaenoic acid (DHA) has anti-inflammatory properties and is a component of neural tissue that normally accumulates rapidly in the brain during the last trimester of pregnancy.^4^ Children born at less than 29 weeks’ gestation receive approximately one-third of the estimated in utero dose of DHA and have lower levels of DHA in their neural tissue than children born at term.^4^ In a previous randomized clinical trial,^5^ we hypothesized that meeting the in utero requirement of DHA during neonatal hospitalization would decrease the risk of BPD and improve IQ in children born extremely preterm. Contrary to expectations, in our N-3 Fatty Acids for Improvement in Respiratory Outcomes (N3RO) randomized trial,^5^ we found that DHA supplementation of children born at less than 29 weeks’ gestation increased the risk of BPD from 43.9% to 49.1%. Although BPD has been found to be associated with poorer cognitive outcomes,^6,7,8,9^ our assessment at 5 years’ corrected age demonstrated a 3.45-point increase in mean full-scale IQ with DHA supplementation.^10^ To our knowledge, few other studies have tested the association of in utero DHA dose with the neurodevelopment of infants born earlier than 29 weeks. Those study results were inconsistent possibly because they conducted assessments at younger ages, for which measurements less reliably predict IQ.^11,12^

Findings from the N3RO trial may create a dilemma for clinicians in the neonatal unit concerning how best to balance perceived risks and benefits associated with high-dose DHA supplementation. BPD occurs among approximately half of children born at less than 29 weeks’ gestation and is a major challenge for clinical practice. However, high-dose DHA supplementation provides one of the few treatments in the neonatal unit found to improve IQ and potentially minimize the longer-term burden facing these children. Such a benefit is particularly compelling given that cognitive outcomes of children born at less than 29 weeks’ gestation have had little improvement over the last 30 years.^1^

In this cohort study, we conducted an exploratory mediation analysis of results from the N3RO trial with the aim of untangling the opposing outcomes associated with DHA supplementation for BPD and IQ in children born at less than 29 weeks’ gestation. In particular, we sought to quantify the indirect effect the DHA-related increase in BPD risk had on the IQ benefit and the direct effect of DHA supplementation on IQ independent of earlier effects on BPD risk.

## Methods

The original N3RO trial and its 5-year follow-up were approved by the Human Research Ethics Committee at each participating hospital (lead hospital, Women’s and Children’s Health Network [WHCN]). Written informed consent was obtained from guardians prior to randomization and before the appointment at 5 years in the N3RO trial. This cohort study’s mediation analysis was also approved by the WCHN Human Research Ethics Committee as the lead hospital with a waiver of consent based on a public interest case (noting that aims were closely related to the original trial aims of determining effects of DHA on BPD and IQ). This study followed the A Guideline for Reporting Mediation Analyses (AGReMA) statement for reporting mediation analyses of randomized trials and observational studies.^16^

### N3RO Trial

This investigation used data collected in the N3RO trial, a multicenter, blinded, parallel-group randomized clinical trial designed to evaluate the effects of DHA supplementation on BPD and IQ in children born extremely preterm. The methods of the trial have been detailed elsewhere.^5,10,13,14^ Briefly, 1273 children born at less than 29 weeks’ gestation were randomized in a 1:1 ratio to receive an enteral DHA emulsion (60 mg/kg/d) or a control emulsion without DHA from the first 3 days of enteral feeds until 36 weeks’ postmenstrual age or discharge home, whichever occurred first. Randomization was stratified by gestational age at birth (<27 weeks and 27 to <29 weeks), sex, and hospital (13 institutions across Australia, New Zealand, and Singapore), with children from a multiple birth randomized individually. Children were assessed for physiological BPD at 36 weeks’ postmenstrual age (the primary outcome of the initial phase of the trial), determined by requirements for supplemental oxygen or respiratory support with an assessment of oxygen saturation.^15^ All randomized and surviving children from the 5 highest-recruiting hospitals in Australia (656 children) were then invited to undergo an assessment using the *Wechsler Preschool and Primary Scale of Intelligence, 4th Edition* at 5 years’ corrected age to determine full-scale IQ (the primary outcome of the follow-up phase); 5 hospitals participated in this phase of the trial based on statistical power considerations. Recruitment into the N3RO trial was done from June 2012 to September 2015, with 5-year IQ assessments performed between August 2018 and May 2021.

### Outcomes of Interest and Assumed Mediation Model

In this analysis, we divide the previously reported total effect of DHA supplementation on IQ^10^ into interventional direct and indirect effects,^17^ with BPD as the presumed mediating variable. The interventional direct effect describes the association between DHA supplementation and IQ that is independent of earlier effects on BPD. Equivalently, it is the estimated benefit to IQ associated with DHA supplementation if hypothetically we could intervene to eliminate the excess risk of BPD with DHA supplementation (ie, mean IQ under DHA with hypothetical BPD intervention vs mean IQ under control). The interventional indirect effect describes the amount of change in the IQ benefit that was associated with the DHA-related effect on BPD, or equivalently, the additional benefit to IQ associated with intervening to eliminate the excess risk of BPD under DHA supplementation (ie, mean IQ under DHA with hypothetical BPD intervention vs mean IQ under DHA). In addition to dividing the total effect of DHA supplementation on IQ into these direct and indirect effects, we also sought to describe the association between BPD and IQ in preliminary analyses.

**Figure 1.**
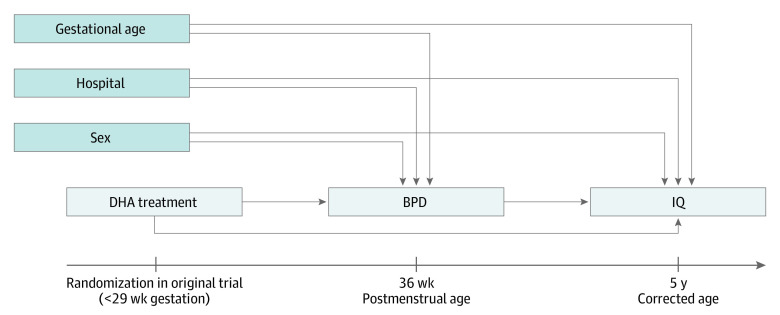
Directed Acyclic Graph Summarizing Assumed Causal Structure BPD indicates bronchopulmonary dysplasia; DHA, docosahexaenoic acid.

The conceptual model guiding the mediation analysis is shown in Figure 1. The effects of DHA supplementation on BPD and IQ were assumed to be unconfounded given the randomized design of the original study, with treatment group defined according to randomization, irrespective of compliance. Conversely, the BPD-IQ association was assumed to be subject to confounding by gestational age at birth, hospital, and sex. Gestational age at birth was dichotomized as less than 27 weeks or 27 to 28 weeks for consistency with its use in stratifying the randomization and earlier analyses estimating treatment effects on BPD and IQ. As a widely established risk factor associated with BPD and IQ deficits, gestational age at birth was considered the main confounder in this analysis.^1,18,19^ Hospital was included as an indicator of clinical care and demographic and medical characteristics of the children, with the risk of BPD and mean IQ observed to vary substantially across the 5 hospitals involved in the follow-up phase of the N3RO trial. Finally, sex was chosen because males born extremely preterm have been found to have a higher risk of BPD^19,20^ and more pronounced cognitive impairment^21,22,23^ than females. Other potential confounders (eg, caffeine therapy, antenatal steroids, postnatal steroids, and severe infant brain injury) were considered but were universally used (ie, caffeine) or not associated with BPD and IQ in the N3RO data set. Included confounders were measured at baseline and used to stratify the randomization in the original study and so were strictly balanced between DHA and control groups. Primary analyses were performed under an assumption of no residual confounding, with the robustness of findings to this assumption explored in sensitivity analyses.

Owing to the blinded nature of the original trial, proposed confounders in the causal diagram and BPD at 36 weeks’ postmenstrual age were ascertained in a fully blinded manner. The trial statistical team (T.R.S. and J.M.B.) and 9 parents representing 11 children (5 children in the DHA group and 6 children in the control group) were unblinded after the analysis of BPD data. Except for these individuals, all participants, study personnel, and outcome assessors remained blinded to group allocation through completion of the IQ assessments at 5 years’ corrected age.

### Statistical Analysis

Analyses included all randomized and surviving children from 5 Australian hospitals involved in the 5-year follow-up phase of the trial (656 children). Statistical calculations were performed using Stata statistical software version 17 (StataCorp), with a 2-tailed *P* < .05 considered to be statistically significant. Data were analyzed from November 2022 to February 2023.

Preliminary analyses were undertaken to describe the total effect of DHA supplementation on IQ, as reported previously,^10^ and the association between BPD and IQ under DHA supplementation. Consistent with earlier analyses, the total effect of DHA supplementation on IQ was estimated via linear regression, with adjustment for stratification variables^24^ and using generalized estimating equations to account for multiple births. An independence working correlation structure was used to produce a treatment effect estimate with a child-level interpretation.^25^ The association between BPD and IQ under DHA supplementation was estimated using linear regression with adjustment for confounding variables and allowing for 2-way interactions between confounders and BPD for flexibility. However, a BPD by hospital interaction term was not included due to sample size constraints. The mean change in IQ associated with BPD was estimated from the model using *g* computation.

For the mediation analysis, we used a simulation-based *g* computation approach to divide the total effect of DHA supplementation on IQ into interventional direct and indirect effects.^17,26^ The approach involved simulating BPD values for participants in the DHA group assuming that their risk of BPD was lowered to match that of the control group (1000 Monte Carlo draws each), then simulating IQ values for these participants using updated BPD values. The mean simulated IQ was used to derive direct and indirect effects, with CIs for estimates obtained using bootstrapping (200 samples, percentile method, and resampling of mothers to account for multiple births). The approach required specification of a regression model for BPD and another for IQ, with both models informed by the directed acyclic graph in Figure 1. BPD was modeled using logistic regression, with treatment group, stratification variables, and 2-way interactions between each stratification variable and treatment group included as factors. IQ was modeled using linear regression, with treatment group, BPD, confounding variables, and 2-way interaction terms involving treatment group included as factors. In both regression models, the interaction term involving hospital was omitted due to sample size constraints.

A complicating factor for analysis was the extent of missing data for IQ. As the probability of missing data depended on observed characteristics in the data set, multiple imputation implemented under a missing-at-random assumption was used to address missing data. Imputation was performed separately by randomization group using chained equations,^27^ with linear and logistic models used to impute missing IQ and BPD values, respectively. A rich set of auxiliary variables was incorporated into models for IQ and BPD to minimize the potential for bias under a missing-at-random assumption. Given that children from a multiple birth were randomized individually, all observations were considered independent in the imputation model.^28^ A total of 100 complete data sets were generated, with results combined across data sets using Rubin rules. Additional details on imputation methods (including auxiliary variables) are provided in the eAppendix in Supplement 1.

The robustness of findings to assumptions about the missing data, underlying causal model, and approach to defining BPD was investigated in sensitivity analyses. For missing data, we considered missing not at random mechanisms via pattern mixture models, with IQ scores assumed to be 3 points lower or 3 points higher in children with missing data compared with children who had observed data. This difference was viewed as a very large departure from the 0-point difference expected under a missing-at-random assumption. We did not undertake sensitivity analyses for the imputation of BPD given that this measure had a low level of missing data. For the assumed causal model, we considered a sensitivity analysis with small for gestational age included as an additional confounder of the BPD-IQ association. This confounder was defined as a birthweight less than the 10th percentile given sex and gestational age.^29^ Small for gestational age is a known risk factor associated with BPD,^19^ yet its association with IQ is less compelling.^1^ Additionally, we were wary of estimation issues with sex and gestational age (measures used in its derivation) that were already included as confounders. Lastly, we performed a sensitivity analysis with BPD defined according to clinical criteria (clinical management with supplemental oxygen or respiratory support at 36 weeks’ postmenstrual age) instead of the physiological definition used in the primary analysis.

## Results

**Figure 2.**
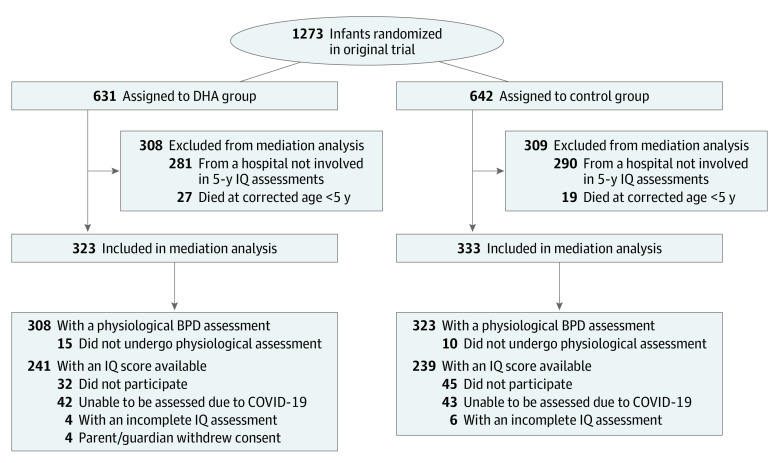
Flowchart for Original Trial and Data Used in Mediation Analysis BPD indicates bronchopulmonary dysplasia; DHA, docosahexaenoic acid.

Of 656 children involved in this analysis (mean [SD] gestational age at birth, 26.8 [1.4] weeks; 346 males [52.7%]), 631 children (96.2%) had a physiological BPD assessment and 480 children (73.2%) had an IQ score available (Figure 2). There were 323 children with DHA supplementation and 333 children in the control group. Baseline characteristics were similar between treatment groups (Table 1), as was compliance with the assigned emulsion, with a mean (SD) of 92.0% (10.5%) of scheduled doses administered to children in the DHA group and 91.7% (10.7%) to children in the control group. At 36 weeks postmenstrual age, the mean (SD) level of DHA in whole blood in the DHA vs control group was 3.9% (0.7%) vs 2.5% (0.5%) of total fatty acids (*P* < .001), or 58.7% higher in the DHA group than the control group. In 656 children included in the mediation analysis, the risk of BPD was increased by 6.9 percentage points in those receiving DHA compared with those in the control group (increase from 143 children in the control group [42.8%] to 160 children in the DHA group [49.7%] based on imputed data). This was a similar increase to that observed in all children in the original phase of the N3RO trial (increase from 43.9% to 49.1%).

**Table 1.** Baseline Characteristics of Children and Parents

Characteristic	Participants, No. (%) (N = 656)	
	DHA group (n = 323)	Control group (n = 333)
**Children**		
Gestational age at birth, mean (SD), wk	26.8 (1.4)	26.8 (1.5)
Gestational age at birth <27 wk	160 (49.5)	157 (47.1)
Sex		
Female	151 (46.7)	159 (47.7)
Male	172 (53.3)	174 (52.3)
Singleton birth	230 (71.2)	238 (71.5)
Birthweight, mean (SD), g	932 (230)	934 (220)
Birthweight small for gestational age	45 (13.9)	40 (12.0)
Age at randomization, median (IQR), d	3.0 (2.0-4.0)	3.0 (2.0-4.0)
DHA in whole blood, mean (SD), % of total fatty acids^a^	2.8 (0.8)	2.8 (0.8)
**Parents** ^b^		
Maternal age, mean (SD), y	30.4 (5.7)	29.9 (5.7)
Mother completed secondary education	219/302 (72.5)	238/314 (75.8)
Mother underwent cesarean delivery	190 (58.8)	186 (55.9)
Father or other parent completed secondary education	194/292 (66.4)	199/300 (66.3)

Abbreviation: DHA, docosahexaenoic acid.

^a^ Data were missing for 1 child in the DHA group and 1 child in the control group.

^b^ Maternal and paternal baseline characteristics are summarized at the child level (ie, parents were counted multiple times if they had multiple children).

Mean IQ scores were higher among children in the DHA group and lower in children with a diagnosis of BPD (Table 2). Children with BPD had lower IQ scores than those without BPD in both treatment groups, while DHA supplementation was associated with an increase in IQ regardless of BPD status. Descriptively, a diagnosis of BPD was associated with a 3.29-point reduction in mean IQ under DHA supplementation (97.0 points; 95% CI, 94.1 to 100.0 points with BPD vs 93.7 points; 95% CI, 90.5 to 97.0 points without BPD) (Table 2). This mean reduction was not statistically significant with adjustment for confounding (2.90 points; 95% CI, −1.44 to 7.31 points).

**Table 2.** IQ of Children Born at <29 Weeks’ Gestation^a^

IQ measure	DHA group (n = 323)		Control group (n = 333)	
	With BPD (n = 160) (49.7%)	Without BPD (n = 163) (50.3%)	With BPD (n = 143) (42.8%)	Without BPD (n = 190) (57.2%)
Mean (95% CI)	93.7 (90.5-97.0)	97.0 (94.1-100.0)	88.8 (85.1-92.5)	94.2 (91.1-97.3)

Abbreviations: BPD, bronchopulmonary dysplasia; DHA, docosahexaenoic acid.

^a^ Summary statistics were based on 100 multiply imputed data sets; numbers with and without BPD were rounded to the nearest whole number.

As previously reported, mean IQ at 5 years’ corrected age improved from 91.91 points in the control group to 95.36 points in DHA group (mean difference, 3.45 points; 95% CI, 0.38 to 6.53 points).^10^ The separation of this total effect of DHA supplementation into direct and indirect effects via the mediation analysis is summarized in Table 3. With adjustment for confounding, eliminating the excess risk of BPD in the DHA group was associated with an increase in the estimated mean (SD) IQ under DHA supplementation from 95.36 (95% CI, 93.18 to 97.57) points to 95.53 (95% CI, 93.33 to 97.74) points. This leads to a direct effect estimate of 3.62 points (95% CI, 0.55 to 6.81 points) and a nonsignificant indirect effect of −0.17 points (95% CI, −0.62 to 0.13 points). That is, most of the effect of DHA supplementation on IQ was direct, with earlier effects on BPD only slightly diminishing the observed IQ benefit.

**Table 3.** Separation of Total Effect of DHA Supplementation on IQ Into Direct and Indirect Effects

Analysis	Change in IQ, points (95% CI)		
	Total effect of DHA (control)	Direct effect	Indirect effect
Primary analysis	3.45 (0.38 to 6.53)	3.62 (0.55 to 6.81)	−0.17 (−0.62 to 0.13)
Sensitivity analysis			
IQ imputed 3 points higher	3.39 (0.26 to 6.52)	3.56 (0.53 to 6.72)	−0.17 (−0.63 to 0.13)
IQ imputed 3 points lower	3.50 (0.34 to 6.67)	3.67 (0.62 to 6.87)	−0.17 (−0.63 to 0.14)
IQ imputed 3 points lower in DHA and 3 points higher in control group	1.37 (−1.76 to 4.51)	1.54 (−1.49 to 4.70)	−0.17 (−0.63 to 0.13)
IQ imputed 3 points higher in DHA and 3 points lower in control group	5.52 (2.36 to 8.69)	5.69 (2.64 to 8.89)	−0.17 (−0.63 to 0.14)
Small for gestational age included as confounder^a^	3.42 (0.38 to 6.46)	3.58 (0.52 to 6.66)	−0.17 (−0.62 to 0.18)
BPD defined using clinical criteria	3.45 (0.38 to 6.53)	3.59 (0.53 to 6.78)	−0.15 (−0.57 to 0.13)

Abbreviations: BPD, bronchopulmonary dysplasia; DHA, docosahexaenoic acid.

^a^ The change in the total effect in this sensitivity analysis was due to refitting the imputation model for compatibility with the mediation analysis.

Table 3 also presents the results of sensitivity analyses around the assumed missing data mechanism, underlying causal model, and definition of BPD. Overall, there was little difference in the indirect effect estimate across these analyses (range, −0.17 points; 95% CI, −0.62 to 0.13 points in the primary analysis to −0.15 points; 95% CI, −0.57 to 0.13 points with BPD defined using clinical criteria). In turn, the direct effect estimate was relatively consistent across analyses, except for more extreme scenarios in which IQ scores were imputed to be 6 points higher in 1 treatment group than the other. Total and direct effects were larger in magnitude when children in the DHA group were imputed to have higher IQ scores than children in the control group (and similarly, smaller in magnitude when they were imputed to have lower scores).

## Discussion

We previously found that providing the in utero DHA requirement to children born at less than 29 weeks’ gestation was associated with improved IQ at 5 years’ corrected age, despite an increased risk of BPD at 36 weeks’ postmenstrual age.^5,10^ In this cohort study’s mediation analysis, we found that the increase in BPD risk from neonatal DHA supplementation was not associated with a material decrease in its benefit to IQ. More formally, the nonsignificant interventional indirect effect estimate suggests that if all children received DHA supplementation, a hypothetical intervention to lower the risk of BPD to that seen under control conditions may be associated with little additional improvements to IQ.

The limited evidence for an indirect effect on IQ via BPD suggests that the mechanisms by which DHA supplementation was associated with improved IQ may differ from those for BPD. While the exact biological mechanisms by which DHA may be associated with IQ are not known, a substantial body of work indicates that DHA has structural and functional roles in neural tissues, including in synaptogenesis and neurite growth, that may contribute to cognitive benefits.^30^ On the other hand, the postulated mechanisms of action for DHA on BPD include the reduction of oxidative stress and inflammation underlying the pathogenesis of BPD.^31^ However intervention trials have not supported the association of high-dose DHA with a beneficial BPD outcome in children born very preterm, with a 2023 meta-analysis^31^ finding no overall benefit and a moderate increase in BPD risk in findings from trials with a standardized definition of BPD as was used in the N3RO trial. While more work is needed to understand the differential mechanisms by which DHA may be associated negatively with BPD and positively with IQ, our analysis suggests that if clinicians decide to supplement infants born very preterm with high-dose DHA, any resulting increase in BPD risk may not be associated with meaningful reductions in beneficial outcomes for IQ.

### Strengths and Limitations

This cohort study’s mediation analysis was based on data from a large, rigorously designed, blinded trial with high levels of compliance and standardized measures of BPD and IQ. However, our study has several limitations. The original trial had higher than expected rates of attrition by 5 years’ corrected age owing to the COVID-19 pandemic; however, reassuringly, the magnitude of the indirect effect of DHA supplementation via BPD appeared robust to specific assumptions made about missing data. The accuracy of indirect and direct effect estimates relied on appropriate specification of the underlying causal model linking DHA supplementation, BPD and IQ together. Given the randomized design of the original trial, our concern was restricted to confounders of the BPD-IQ association, and while effort was made to incorporate key confounders, it is not possible to rule out the effects of residual confounding. Additionally, it should be noted that this analysis did not address the trade-off between an increased risk of BPD and improved IQ associated with high-dose DHA supplementation. Although we did not observe adverse health signals, including for respiratory-related hospitalizations, from parent reports at the 5-year assessment, further investigation is needed to fully assess the association of the modest increase in BPD risk from DHA with long-term pulmonary function.

## Conclusions

This cohort study found that the increased risk of BPD from neonatal DHA supplementation in children born at less than 29 weeks’ gestation was not associated with a decrease in the positive effect of DHA on IQ. This finding suggests that clinicians may be able to supplement children born preterm with high-dose DHA without meaningful reductions in IQ benefit.

## Notes

Supplement 1. **eAppendix.** Multiple Imputation Approach

Supplement 2.
Data Sharing Statement

## References

[1] Twilhaar ES, Wade RM, de Kieviet JF, van Goudoever JB, van Elburg RM, Oosterlaan J. Cognitive outcomes of children born extremely or very preterm since the 1990s and associated risk factors: a meta-analysis and meta-regression. JAMA Pediatr. 2018;172(4):361-367. doi:10.1001/jamapediatrics.2017.5323 29459939 PMC5875339

[2] Brydges CR, Landes JK, Reid CL, Campbell C, French N, Anderson M. Cognitive outcomes in children and adolescents born very preterm: a meta-analysis. Dev Med Child Neurol. 2018;60(5):452-468. doi:10.1111/dmcn.13685 29453812

[3] Allotey J, Zamora J, Cheong-See F, . Cognitive, motor, behavioural and academic performances of children born preterm: a meta-analysis and systematic review involving 64 061 children. BJOG. 2018;125(1):16-25. doi:10.1111/1471-0528.14832 29024294

[4] Martinez M. Tissue levels of polyunsaturated fatty acids during early human development. J Pediatr. 1992;120(4 Pt 2):S129-S138. doi:10.1016/S0022-3476(05)81247-8 1532827

[5] Collins CT, Makrides M, McPhee AJ, . Docosahexaenoic acid and bronchopulmonary dysplasia in preterm infants. N Engl J Med. 2017;376(13):1245-1255. doi:10.1056/NEJMoa1611942 28355511

[6] Schmidt B, Asztalos EV, Roberts RS, Robertson CM, Sauve RS, Whitfield MF; Trial of Indomethacin Prophylaxis in Preterms (TIPP) Investigators. Impact of bronchopulmonary dysplasia, brain injury, and severe retinopathy on the outcome of extremely low-birth-weight infants at 18 months: results from the trial of indomethacin prophylaxis in preterms. JAMA. 2003;289(9):1124-1129. doi:10.1001/jama.289.9.1124 12622582

[7] Jensen EA, Dysart K, Gantz MG, . The diagnosis of bronchopulmonary dysplasia in very preterm infants. an evidence-based approach. Am J Respir Crit Care Med. 2019;200(6):751-759. doi:10.1164/rccm.201812-2348OC 30995069 PMC6775872

[8] Jobe AH, Steinhorn R. Can we define bronchopulmonary dysplasia? J Pediatr. 2017;188:19-23. doi:10.1016/j.jpeds.2017.06.064 28705654

[9] Gilfillan M, Bhandari A, Bhandari V. Diagnosis and management of bronchopulmonary dysplasia. BMJ. 2021;375:n1974. doi:10.1136/bmj.n1974 34670756

[10] Gould JF, Makrides M, Gibson RA, . Neonatal docosahexaenoic acid in preterm infants and intelligence at 5 years. N Engl J Med. 2022;387(17):1579-1588. doi:10.1056/NEJMoa2206868 36300974

[11] Guillot M, Synnes A, Pronovost E, . Maternal high-dose DHA supplementation and neurodevelopment at 18-22 months of preterm children. Pediatrics. 2022;150(1):e2021055819. doi:10.1542/peds.2021-055819 35652296

[12] Hewawasam E, Collins CT, Muhlhausler BS, . DHA supplementation in infants born preterm and the effect on attention at 18 months’ corrected age: follow-up of a subset of the N3RO randomised controlled trial. Br J Nutr. 2021;125(4):420-431. doi:10.1017/S0007114520002500 32660658

[13] Collins CT, Gibson RA, Makrides M, ; N3RO Investigative Team. The N3RO trial: a randomised controlled trial of docosahexaenoic acid to reduce bronchopulmonary dysplasia in preterm infants < 29 weeks’ gestation. BMC Pediatr. 2016;16:72. doi:10.1186/s12887-016-0611-0 27250120 PMC4896378

[14] Gould JF, Makrides M, Sullivan TR, . Protocol for assessing whether cognition of preterm infants <29 weeks’ gestation can be improved by an intervention with the omega-3 long-chain polyunsaturated fatty acid docosahexaenoic acid (DHA): a follow-up of a randomised controlled trial. BMJ Open. 2021;11(2):e041597. doi:10.1136/bmjopen-2020-041597 33550243 PMC7925903

[15] Walsh MC, Yao Q, Gettner P, ; National Institute of Child Health and Human Development Neonatal Research Network. Impact of a physiologic definition on bronchopulmonary dysplasia rates. Pediatrics. 2004;114(5):1305-1311. doi:10.1542/peds.2004-0204 15520112

[16] Lee H, Cashin AG, Lamb SE, ; AGReMA group. A guideline for reporting mediation analyses of randomized rials and observational studies: the AGReMA statement. JAMA. 2021;326(11):1045-1056. doi:10.1001/jama.2021.14075 34546296 PMC8974292

[17] Vansteelandt S, Daniel RM. Interventional effects for mediation analysis with multiple mediators. Epidemiology. 2017;28(2):258-265. doi:10.1097/EDE.0000000000000596 27922534 PMC5289540

[18] Stoll BJ, Hansen NI, Bell EF, ; Eunice Kennedy Shriver National Institute of Child Health and Human Development Neonatal Research Network. Neonatal outcomes of extremely preterm infants from the NICHD Neonatal Research Network. Pediatrics. 2010;126(3):443-456. doi:10.1542/peds.2009-2959 20732945 PMC2982806

[19] Trembath A, Laughon MM. Predictors of bronchopulmonary dysplasia. Clin Perinatol. 2012;39(3):585-601. doi:10.1016/j.clp.2012.06.014 22954271 PMC3443959

[20] Bose C, Van Marter LJ, Laughon M, ; Extremely Low Gestational Age Newborn Study Investigators. Fetal growth restriction and chronic lung disease among infants born before the 28th week of gestation. Pediatrics. 2009;124(3):e450-e458. doi:10.1542/peds.2008-3249 19706590 PMC2891899

[21] Linsell L, Malouf R, Morris J, Kurinczuk JJ, Marlow N. Prognostic factors for poor cognitive development in children born very preterm or with very low birth weight: a systematic review. JAMA Pediatr. 2015;169(12):1162-1172. doi:10.1001/jamapediatrics.2015.2175 26457641 PMC5122448

[22] Kuban KC, Joseph RM, O’Shea TM, ; Extremely Low Gestational Age Newborn (ELGAN) Study Investigators. Girls and boys born before 28 weeks gestation: risks of cognitive, behavioral, and neurologic outcomes at age 10 years. J Pediatr. 2016;173:69-75.e1. doi:10.1016/j.jpeds.2016.02.048 27004675 PMC4884461

[23] Linsell L, Johnson S, Wolke D, . Cognitive trajectories from infancy to early adulthood following birth before 26 weeks of gestation: a prospective, population-based cohort study. Arch Dis Child. 2018;103(4):363-370. doi:10.1136/archdischild-2017-313414 29146572 PMC5890637

[24] Kahan BC, Morris TP. Improper analysis of trials randomised using stratified blocks or minimisation. Stat Med. 2012;31(4):328-340. doi:10.1002/sim.4431 22139891

[25] Yelland LN, Sullivan TR, Pavlou M, Seaman SR. Analysis of randomised trials including multiple births when birth size is informative. Paediatr Perinat Epidemiol. 2015;29(6):567-575. doi:10.1111/ppe.12228 26332368 PMC4847643

[26] Moreno-Betancur M, Moran P, Becker D, Patton GC, Carlin JB. Mediation effects that emulate a target randomised trial: simulation-based evaluation of ill-defined interventions on multiple mediators. Stat Methods Med Res. 2021;30(6):1395-1412. doi:10.1177/0962280221998409 33749386 PMC8371283

[27] White IR, Royston P, Wood AM. Multiple imputation using chained equations: Issues and guidance for practice. Stat Med. 2011;30(4):377-399. doi:10.1002/sim.4067 21225900

[28] Sullivan TR, Yelland LN, Moreno-Betancur M, Lee KJ. Multiple imputation for handling missing outcome data in randomized trials involving a mixture of independent and paired data. Stat Med. 2021;40(27):6008-6020. doi:10.1002/sim.9166 34396577

[29] Beeby PJ, Bhutap T, Taylor LK. New South Wales population-based birthweight percentile charts. J Paediatr Child Health. 1996;32(6):512-518. doi:10.1111/j.1440-1754.1996.tb00965.x 9007782

[30] Basak S, Mallick R, Duttaroy AK. Maternal docosahexaenoic acid status during pregnancy and its impact on infant neurodevelopment. Nutrients. 2020;12(12):3615. doi:10.3390/nu12123615 33255561 PMC7759779

[31] Marc I, Boutin A, Pronovost E, . Association between enteral supplementation with high-dose docosahexaenoic acid and risk of bronchopulmonary dysplasia in preterm infants: a systematic review and meta-analysis. JAMA Netw Open. 2023;6(3):e233934. doi:10.1001/jamanetworkopen.2023.3934 36943265 PMC10031388

