# Supplementary material for: Mediation Analysis to Untangle Opposing Associations of High-Dose Docosahexaenoic Acid With IQ and Bronchopulmonary Dysplasia in Children Born Preterm
Source: JAMA Netw Open. 2023 Jun 9;6(6):e2317870. doi: 10.1001/jamanetworkopen.2023.17870 (PMC10257101; doi:10.1001/jamanetworkopen.2023.17870)
Supplement: Supplement 1. — eAppendix. Multiple Imputation Approach [file jamanetwopen-e2317870-s001.pdf]

Supplemental Online Content

Sullivan TR, Gould JF, Bednarz JM, et al. Mediation analysis to untangle opposing associations of high-dose docosahexaenoic acid with IQ and bronchopulmonary dysplasia in children born preterm. *JAMA Netw Open*. 2023;6(6):e2317870. doi:10.1001/jamanetworkopen.2023.17870

eAppendix. Multiple Imputation Approach

This supplemental material has been provided by the authors to give readers additional information about their work.

© 2023 Sullivan TR et al. *JAMA Network Open.*

eAppendix. Multiple Imputation Approach

Missing data on full-scale IQ (27% missing) and BPD (4% missing) were addressed using multiple imputation implemented under a missing at random assumption. Imputation was performed separately by randomized group using fully conditional specification (also known as chained equations), with 100 complete datasets generated for analysis. Since children from a multiple birth were randomized individually, all observations were considered independent in the imputation model. Missing IQ scores were imputed using linear regression, while missing BPD values were imputed using logistic regression. As well as analysis model variables (gestational age, sex and hospital), conditional regression models included auxiliary variables associated with IQ and BPD values and/or their missingness (to improve the prediction of missing values and the plausibility of a missing at random assumption). Where continuous auxiliary variables were included in the imputation model, a linear relationship between the auxiliary variable and the link function of the incomplete outcome was assumed. Auxiliary variables for IQ included birth by caesarean section, maternal race, maternal age, smoking during pregnancy, maternal highest level of education, baseline DHA level in whole blood, hospital length of stay, necrotizing enterocolitis, grade 3 or 4 intraventricular haemmorhage, stage ≥3 retinopathy of prematurity, sepsis, cerebral cystic formation, antenatal corticosteroids and singleton birth. Auxiliary variables for BPD included days of supplemental oxygen to 36 week's gestation, type of respiratory support at 36 weeks' gestation (defining the need for a physiological challenge or oximetry) and singleton birth. The imputation model was fitted using the mi impute chained command in Stata version 17, with 50 burn-in iterations used in each cycle of the procedure. In calculating bootstrap confidence intervals for the direct and indirect effects, bootstrapping was performed within each imputed dataset rather than prior to imputation.

© 2023 Sullivan TR et al. *JAMA Network Open.*
