# Supplementary material for: Mediation Analysis to Untangle Opposing Associations of High-Dose Docosahexaenoic Acid With IQ and Bronchopulmonary Dysplasia in Children Born Preterm
Source: JAMA Netw Open. 2023 Jun 9;6(6):e2317870. doi: 10.1001/jamanetworkopen.2023.17870 (PMC10257101; doi:10.1001/jamanetworkopen.2023.17870)
Supplement: Supplement 2. — Data Sharing Statement [file jamanetwopen-e2317870-s002.pdf]

Data Sharing Statement

Sullivan. Mediation Analysis to Untangle Opposing Associations of High-Dose Docosahexaenoic Acid With IQ and Bronchopulmonary Dysplasia in Children Born Preterm. *JAMA Netw Open*. Published online June 9, 2023. doi:10.1001/jamanetworkopen.2023.17870

Data
Data available: Yes
Data types: Other (please specify)

Supporting Documents
Document types: None

Additional Information: De-identified data will be made available, upon reasonable request to the authors and approval of the governing institutional review board.
How to access data: Requests should be sent to Thomas Sullivan thomas.sullivan@sahmri.com and Maria Makrides maria.makrides@sahmri.com
When available: With publication

Additional Information
Who can access the data: researchers whose proposed use of the data has been approved
Types of analyses: For any approved purpose.
Mechanisms of data availability: After approval of a proposal and with a signed data access agreement.

© 2023 Sullivan TR et al. *JAMA Network Open*.
